# Significance of Measurable Residual Disease in Patients Undergoing Allogeneic Hematopoietic Cell Transplantation for Acute Myeloid Leukemia

**DOI:** 10.3390/cells14040290

**Published:** 2025-02-15

**Authors:** Margery Gang, Megan Othus, Roland B. Walter

**Affiliations:** 1 Hematology and Oncology Fellowship Program, Fred Hutchinson Cancer Center, University of Washington, Seattle, WA 98109, USA; mgang@uw.edu; 2 Public Health Sciences Division, Fred Hutchinson Cancer Center, Seattle, WA 98109, USA; mothus@fredhutch.org; 3 Translational Science and Therapeutics Division, Fred Hutchinson Cancer Center, Seattle, WA 98109, USA; 4 Division of Hematology and Oncology, Department of Medicine, University of Washington, Seattle, WA 98195, USA; 5 Department of Laboratory Medicine and Pathology, University of Washington, Seattle, WA 98195, USA

**Keywords:** acute myeloid leukemia, allogeneic, biomarker, hematopoietic cell transplantation, measurable residual disease, prognostication

## Abstract

Allogeneic hematopoietic cell transplantation (HCT) remains an important curative-intent treatment for many patients with acute myeloid leukemia (AML), but AML recurrence after allografting is common. Many factors associated with relapse after allogeneic HCT have been identified over the years. Central among these is measurable (“minimal”) residual disease (MRD) as detected by multiparameter flow cytometry, quantitative polymerase chain reaction, and/or next-generation sequencing. Demonstration of a strong, independent prognostic role of pre- and early post-HCT MRD has raised hopes MRD could also serve as a predictive biomarker to inform treatment decision-making, with emerging data indicating the potential value to guide candidacy assessment for allografting as a post-remission treatment strategy, the selection of conditioning intensity, use of small molecule inhibitors as post-HCT maintenance therapy, and preemptive infusion of donor lymphocytes. Monitoring for leukemia recurrence after HCT and surrogacy for treatment response are other considerations for the clinical use of MRD data. In this review, we will outline the current landscape of MRD as a biomarker for patients with AML undergoing HCT and discuss areas of uncertainty and ongoing research.

## 1. Introduction

Allogeneic hematopoietic cell transplantation (HCT) remains an important cornerstone of acute myeloid leukemia (AML) therapy [1,2,3,4,5,6]. Unfortunately, AML recurrence after allografting is common, constituting the major cause of HCT failure. Identifying patients at high risk of relapse is crucial to refine prognostication and decision-making about the benefits and risks of allografting as well as to implement risk-adapted therapeutic interventions. A myriad of pre- and early post-HCT factors associated with relapse after allogeneic HCT have been identified over the years. Of these, measurable (or “minimal”) residual disease (MRD) has garnered significant interest. Demonstrating a strong, independent prognostic role of MRD has raised interest in its potential use as a predictive biomarker to inform treatment decision-making, for disease monitoring, and as a surrogate efficacy-response biomarker. In this review, we will summarize the existing knowledge regarding the significance of MRD for patients with AML undergoing allografting and highlight areas of uncertainty and the need for additional data.

## 2. Methodologies for MRD Testing

Initial efforts to quantify MRD date back 5 decades [7]. Observing high relapse rates fueled the idea that residual AML cells persist below the cytomorphological detection limit after seemingly successful chemotherapy. Further impetus to enumerate such cells came from the demonstration of a quantitative relationship between leukemia cell burdens and the survival of experimental animals [7]. However, genetic heterogeneity and clonal complexity have posed challenges in establishing AML MRD assays, and only a subset of disease entities (e.g., acute promyelocytic leukemia [APL] or core-binding factor [*CBF*] leukemias) is characterized by pathogenetically pivotal, canonical mutations. Consequently, several technologies that detect either immunophenotypic abnormalities or genetic/molecular changes of the neoplastic cells have been developed in parallel for MRD testing [8,9]. Current assays, each with their own advantages and disadvantages, include multiparameter flow cytometry (MFC), quantitative polymerase chain reaction (PCR), and next-generation sequencing (NGS; Table 1). Their primary focus is bone marrow, although peripheral blood and, for molecular testing, circulating cell-free DNA is increasingly used and may yield similar findings [10,11,12]. Single-cell MRD assays are a new development [13,14].

### 2.1. Multiparameter Flow Cytometry (MFC)

MFC identifies AML cells via immunophenotypic abnormalities, specifically leukemia-associated immunophenotypes (LAIPs) and/or cell population(s) deviating from antigen-expression patterns typical of normal or regenerating cells of similar lineage and maturation stage (“different-from-normal [DfN]”) [8,15]. Because of possible immunophenotypic shifts during treatment, combined use of LAIPs and DfN methodologies is recommended. With a comprehensive antibody panel, MFC MRD assays are suitable for >85–90% of all AML cases. The sensitivity varies with the type of phenotypic change(s) relative to normal background cell populations, with fresh samples required for optimal results. Thus, MFC-based MRD assays vary in sensitivity across patients—in most cases, MRD can be detected down to 0.1% when present, but sensitivities can be higher in progressively smaller subsets of patients. Advantages include the ability to identify and quantify an abnormal stem/progenitor cell compartment, estimate hemodilution, distinguish live from dead cells, and identify immunotherapy targets [8,9,15,16,17]. The most notable limitation is the requirement for considerable expertise, as analysis and data assessment typically include subjective elements.

Current methodologies (e.g., number/type of antigens, fluorochromes, data acquisition, and data analysis/interpretation) and performance characteristics of individual assays vary substantially, impeding clinical implementation. Standardization (or, at the very least, harmonization) of technical aspects of MFC MRD tests has proven challenging, but efforts are ongoing [8,15]. Other areas for improvement involve automated data analysis (including automated computational pipelines) and interpretation [18,19] and, possibly, the inclusion of less mature (“leukemia stem cell”) populations. Despite controversies and challenges regarding the definition and isolation of such cell populations, emerging data indicate leukemia stem cell-based MFC MRD testing might surpass and/or complement the performance of conventional assays and refine risk assessment [20,21,22]. As a technological advancement, spectral flow cytometry may enable the evaluation of a greater number of parameters in single analyses and facilitate high-dimensional data acquisition from panels of fluorescent antibodies [23].

### 2.2. Quantitative Polymerase Chain Reaction (PCR)

Quantitative PCR (qPCR)-based MRD assays measure gene rearrangements, chromosomal translocations, or gene mutations defining subsets of AML. By comparing amplified patient RNA to a standard curve derived from cell lines or plasmid DNA, reverse-transcription PCR (RT-PCR) strategies can detect leukemic cells at very low frequencies (10^−5^–10^−6^) [9,24]. This makes RT-PCR valuable for specific mutations and fusion transcripts, including *NPM1*, *FLT3* internal tandem duplication (*FLT3*-ITD), *PML-RARA*, *RUNX1-RUNX1T1*, and *CBFB-MYH11*. Less validated targets include *KMT2A-MLLT3*, *DEK-NUP214*, *BCR-ABL1*, and *WT1*. Assay standardization is less challenging for qPCR compared to other methodologies, simplifying clinical application.

Despite its advantages, qPCR MRD testing has several challenges. Most importantly, it is not applicable to 60–70% of patients with AML as they lack targetable molecular abnormalities [8]. Moreover, some abnormalities amenable to qPCR detection (e.g., mutations in DNA methyltransferase 3A [*DNMT3A*]) may not be informative for relapse risk [25,26,27,28].

Recent techniques, including digital PCR (dPCR), may offer greater accuracy than RT-PCR [29]. As dPCR provides absolute quantification of molecules, standardized assays may be easier to implement clinically than qPCR assays, providing independence from fluctuations in the normalization target and more direct data interpretability [9].

### 2.3. NGS

NGS-based assays have emerged as powerful tools due to their ability to simultaneously detect and quantify multiple genetic mutations in a single assay, reaching sensitivities of 10^−5^–10^−6^ by maximizing sequencing depth (ultrasensitive NGS) or including error correction [30]. Thus, they could theoretically be applied to almost all patients and may be particularly useful for tracking multiple and/or less common mutations.

NGS estimates the mutational burden by quantifying variant allele frequency (VAF) or the proportion of sequencing reads containing the mutant allele relative to the total number of reads [31]. However, mutation persistence is not always prognostically informative, and NGS MRD assays must be interpreted cautiously. For instance, the persistence of *DNMT3A*, *ASXL1*, and *TET2* (“DAT”) mutations, which have been linked to age-related clonal hematopoiesis, is not associated with leukemia relapse or worse survival in patients in morphologic remission [32,33,34].

Conventional NGS assays face challenges with sequencing errors, resulting in both false positive and false negative results approximating 1% [35]. This decreases their sensitivity in detecting low-level genetic variants. To address this shortcoming, several bioinformatic error-suppression/correction approaches (e.g., duplex sequencing) have been developed, including alternative computational approaches and changes to library preparation, to increase the reliability and sensitivity of mutation detection [9,36]. However, even with these improved approaches, significant variability in NGS-based strategies remains, and presently there is no single, uniform standard for NGS MRD assessment [3].

## 3. Limitations of MRD Testing

An ideal MRD assay should accurately and precisely identify the population(s) of leukemia cells that, if left untreated, lead to disease recurrence, while disregarding other leukemia cells that do not cause relapse despite being immunophenotypically, functionally, and/or molecularly abnormal [7,37,38]. Until the characteristics defining these leukemia propagation/relapse-relevant cells are understood, the perfect assay may not exist. Beyond AML biology, limitations in assay methodology, biospecimen sampling, and statistics additionally complicate MRD testing and must be considered [7,16,37,38].

### 3.1. Biospecimen/Sampling Considerations

Like any other medical test, the precision of MRD assays is impacted by sampling volume, as a small blood or marrow sample may not be representative of the whole-body AML burden [7,16,37,38]. Moreover, the heterogeneous distribution of AML cells within the bone marrow microenvironment may lead to “sampling errors”, complicating reliable MRD quantification. Furthermore, unrecognized or incompletely corrected dilution of sample material will lead to falsely-negative or falsely-low results. Leukemia relapses can also occur in sites (e.g., central nervous system, other extramedullary sites) where blood or marrow samples do not correctly estimate true disease burdens. Conceptually paramount, the detection of low levels of residual leukemia cells depends primarily on the sample type and size rather than the sensitivity of the MRD assay per se.

### 3.2. Methodological Considerations

For clinical performance, the theoretical maximum sensitivity and specificity to detect leukemia relapse-relevant AML cells are important characteristics of any MRD assay [7,16,37,38]. Normal and regenerating cells and mutations associated with non-leukemia, clonal hematopoiesis can result in immunophenotypic and molecular “noise” that interferes with detection of residual AML cells. MRD re-testing can decrease the likelihood of incorrect data interpretation. Other characteristics such as reproducibility and repeatability or test-retest reliability (the components of a test’s precision) or replicability are equally important for assay performance [7,16,37,38].

### 3.3. Treatment Considerations

The relationship between MRD test results and relapse is affected by subsequent therapeutic intervention(s). This is particularly relevant for patients receiving allografting given the immunological (“graft-versus-leukemia [GVL]”) effects of allogeneic HCT. Their magnitude is, on average, similar for patients with and those without pre-HCT MRD [39]. Likewise, targeted AML treatments may drive the evolution of a resistant leukemic clone at relapse, thereby affecting MRD testing; e.g., among patients with *FLT3*-ITD positive AML treated with midostaurin, almost half become *FLT3*-ITD negative at the time of disease resistance or progression [40], highlighting the dynamic disease nature and need for ongoing adaptive MRD monitoring for AML relapse.

### 3.4. Statistical Considerations

After allogeneic HCT, key endpoints of interest include time to relapse (arguably of greatest immediate relevance for MRD testing), overall survival, and non-relapse mortality (NRM). While competing risks for each of these time-to-event, censored endpoints are present, they can only imperfectly be accounted for. Estimation of test performance metrics such as sensitivity, specificity, positive predictive value, and negative predictive value need to account for the censored nature of these endpoints [41]. Both prognostic (i.e., is MRD associated with outcome?) and predictive (i.e., can MRD predict a given patient’s outcome?) questions can be asked. Prognostic questions can be answered by a test that generates a *p*-value, the probability that the observed difference occurred by chance. In contrast, predictive questions require alternative measures to evaluate performance such as the area under the receiver operating characteristic curve (AUC) for binary outcomes or the C-statistic for censored data outcomes, which plots the true positive rate against the false positive rate at various thresholds. It is important to note that a test can have a strong prognostic association but a mediocre predictive performance: a statistically significant *p*-value does not imply clinically useful predictive ability [42].

As with other prognostic or predictive tests, the interpretation of MRD data is subject to limitations in statistical properties [37]. MRD assays are commonly reported as binary “negative” or “positive” results, with consensus thresholds defining “positivity” (e.g., ≥0.1% for MFC MRD as per European LeukemiaNet [ELN] AML MRD Working Group recommendations) [8]. The use of binary readouts simplifies continuous outcomes, but this loss of information decreases sensitivity and specificity and prevents the identification of any linear relationship with outcomes [37].

The choice of threshold for MRD can also under-estimate outcome variation between groups: patients with low-level (but positive) MRD tests may have more similar outcomes to those with negative MRD tests than those with high-level positive MRD tests. The use of assay-agnostic, uniform positivity thresholds ignores the diversity of characteristics of individual tests. A recent study comparing four validated MFC assays demonstrated that a universal MRD positivity threshold does not maximize patient stratification; rather, the proportion of patients with any level of MRD or MRD below/above 0.1% varied widely [43]. In addition to an observed non-linear relationship between MRD burden and relapse risk, optimal cut-points to define MRD positivity varied between cohorts/assays, arguing for the need to delineate relevant, individual MRD “negativity/positivity” thresholds using actual assay performance characteristics [43]. Beyond assay methodologies and their precision parameters, optimal thresholds may depend on disease- and treatment-specific contexts, e.g., the time point of MRD assessment, type of therapy, and/or immunophenotypic/molecular heterogeneity across AML subtypes [43]. While perhaps accentuated by the particularly wide methodological differences across current MFC MRD assays, similar principles may apply to molecular MRD testing approaches.

### 3.5. Molecular MRD Biomarker Selection/Interpretation Considerations

Identifying optimal targets for molecular MRD assessment is crucial. Both mutated *NPM1* and *FLT3*-ITD are well-validated targets for patients with baseline *NPM1* and/or *FLT3*-ITD mutations undergoing allografting [44,45]. Pre-HCT detection of fusion transcripts in *KMT2A*-rearranged AML is also strongly prognostic [46,47]. For other mutations, the prognostic significance is less clear. As mentioned, the persistence of “DAT” mutations has no prognostic relevance. A recent retrospective analysis also found no evidence that the detection of *isocitrate dehydrogenase 1* (*IDH1*) mutations in remission before allografting was prognostically informative [48]. In Pre-MEASURE, a large population-based study evaluating the utility of NGS MRD in pre-HCT remission peripheral blood samples, the detection of residual mutations in *IDH1*, *IDH2*, *FLT3*-tyrosine kinase domain (*FLT3*-TKD), or *KIT* had no significant impact on relapse [49]. Notably, in further subset analyses, the persistence of *IDH2* mutations by ultrasensitive error-corrected NGS was associated with an increased risk of relapse and death compared to patients in whom these mutations cleared at the time of allografting [50]. Still, *NPM1* and/or *FLT3*-ITD (which frequently co-occur with *IDH2* mutations) as MRD markers in these patients provided better risk stratification than *IDH2* [50]. As methodological advances improve our ability to detect MRD, the question of the clinical significance of very low VAFs (<2.5%) of either persistent or new variants post-HCT becomes increasingly pertinent [51].

## 4. MRD as Prognostic Biomarker Before and After Allogeneic HCT

Despite these limitations, numerous studies have demonstrated that MRD testing before and/or after HCT allows for effective risk stratification of AML patients receiving allografts. Individuals in MRD-positive remission have a significantly higher likelihood (but not certainty) of relapse and, consequently, shorter survival than those in MRD-negative remission. However, among the latter, leukemia recurrences still occur [7,8,52,53].

### 4.1. Prognostic Significance of Pre-HCT MRD

The prognostic role of pre-HCT MRD has been the most extensively studied. Many retrospective and prospective studies have demonstrated that MRD detected before allografting either by MFC, qPCR, or NGS identifies patients at higher risk of relapse and shorter survival than those testing MRD negative [44,45,49,50,54,55,56,57,58,59,60,61,62]. As a large proportion of the relapses among patients with MRD-positive remission before HCT occur early (e.g., within the first 3 months) after allografting, pre-HCT MRD can serve as a logical indicator of poor short-term outcomes [63]. In addition, pre-HCT MRD is a strong and independent indicator of higher relapse risk and shorter survival in patients who survived 100 days without experiencing disease recurrence [64]. This risk persists even with “conversion” of MRD status; i.e., patients who tested positive for MRD before HCT but were negative for MRD at day +70–100 had worse outcomes compared to those negative at both time points [64].

### 4.2. Prognostic Significance of Post-HCT MRD

Although less well studied, several studies have demonstrated that MRD detected after allogeneic HCT by MFC or NGS has prognostic significance [63,65,66], with rates of relapse approaching 80% in some studies in patients with MRD early following allografting [65]. Among patients transplanted in morphologic remission who survived for at least 100 days without experiencing leukemia relapse, a recent retrospective study found that MRD is uncommon (~1% of patients) around day +100; still, these patients have substantially worse outcomes than those without MRD at that time [64]. Limited data support the prognostic value of MRD before donor lymphocyte infusion (DLI) and at several time points after DLI [67]. Of note, in patients with earlier disease relapse (e.g., within 6 months of HCT), most/all previously seen leukemia-associated markers can be detected in the MRD-positive sample preceding the relapse. Conversely, in patients with later relapse, there is less overlap, suggesting evolution of a subclone with different growth kinetics rather than primary conditioning refractoriness driving AML recurrence [66]. Importantly, even with frequent MRD monitoring, not all post-HCT AML relapses are preceded by an MRD-positive sample. Patients who remain MRD negative on peripheral blood monitoring prior to overt relapse have been found to have worse outcomes compared to those who tested positive for MRD prior to relapse, possibly as a reflection of more aggressive disease kinetics [66].

### 4.3. Prognostic Significance of Serial MRD Testing

Rather than considering pre- or post-HCT MRD in isolation, some studies suggest that peri-HCT MRD dynamics can refine risk assessment [63,64,68]. Such studies show MRD conversion is common: >80% of patients with MRD undergoing myeloablative HCT and >50% of patients with MRD undergoing non-myeloablative conditioning (MAC) HCT clear MRD within 20–40 days after allografting [63]; among patients alive without early relapse, over 90% of patients with MRD will convert to MRD negativity within 70–100 days after allografting [64]. Importantly, while outcomes of MRD “converters” (i.e., patients with MRD-positivity pre-HCT with subsequent MRD-negativity post-HCT [“MRD^pos^/MRD^neg^ patients”]) were better than those who remained MRD positive (i.e., MRD^pos^/MRD^pos^ patients), they remained significantly worse than those testing MRD negative at both time points (i.e., MRD^neg^/MRD^neg^ patients). Notably, although non-MAC regimens were less likely to clear MRD, their impact on outcomes was more significant when they did.

### 4.4. Conclusions on MRD as Prognostic Biomarker for AML Before/After Allogeneic HCT

There is now convincing population-level evidence that MRD, irrespective of the testing methodology, is a valuable prognostic biomarker to inform risk/benefit discussions/assessments for patients with AML undergoing allogeneic HCT across the spectrum of conditioning intensities. The most robust data are from molecular testing for well-defined mutations, including *NPM1* and *FLT3*-ITD [44,49,50,56,60], with increasing use of NGS, reflecting the improved sensitivity and accuracy of newer platforms and ability to standardize assays and interpretation across laboratories. In fact, emerging data suggest carefully optimized NGS MRD assays may be prognostically more informative than standard MFC MRD assays [49,69]. However, there is little evidence to suggest which MRD assay is optimal, particularly when results from different MRD testing methodologies vary. Molecular and MFC MRD have independent and additive prognostic value; detection of MRD with both assays is associated with a high probability of relapse and, conversely, the inability to detect MRD by both assays correlates with a lower probability of relapse [33,57]. As individual assays become further refined, to what degree this will change is an important question to address.

For individual patients, MRD remains an imperfect biomarker: a subset of MRD-negative patients will still relapse while not all patients with detectable MRD will do so. Moreover, our ability to predict post-HCT relapse on an individual patient level remains woefully limited, and MRD data add very little to the accuracy of outcome prediction [70].

## 5. Using MRD to Guide Therapy for Patients Considered for Allogeneic HCT

Conceptually, there are many opportunities for MRD-directed treatment decision-making in AML patients eligible for allogeneic HCT. These include the selection of allografting versus an alternative therapy as the preferred post-remission treatment strategy, additional/intensified therapy to eradicate MRD before allogeneic HCT, choice of conditioning intensity and stem cell source, modulation of immunosuppressive therapy, pre-emptive use of donor lymphocyte infusion (DLI), post-HCT maintenance therapy, and treatment of post-HCT MRD relapse.

### 5.1. Allografting or Alternative Therapy as Preferred Post-Remission Treatment Strategy

Allogeneic HCT is currently the most effective post-remission therapy for AML, with a ~60–65% reduction of relapse risks relative to intensive chemotherapy and/or autologous HCT, regardless of cytogenetic disease risk or MRD status [39,71]. Because of risks from HCT-associated non-relapse morbidity/mortality, allogeneic HCT is typically only considered if the relapse probability without transplant is predicted to exceed 35–40% [3,72]. Hence, allogeneic HCT is not typically offered to patients with cytogenetically/molecularly favorable-risk AML given their high chance of achieving long-term remission with standard chemotherapy alone. However, among those with favorable-risk disease, patients with inadequate clearance of MRD have sufficiently high relapse risk to justify allografting in first morphologic remission [26,73,74,75]. Conversely, clearance of MRD after completion of initial chemotherapy may identify some patients, including those with *NPM1*-mutated AML regardless of *FLT3*-ITD status, intermediate-risk AML without *NPM1* mutation, or *de novo* AML with myelodysplasia-related gene mutations, who do not benefit from immediate allogeneic HCT [76,77,78,79].

### 5.2. Additional/Intensified Therapy to Eradicate MRD Before Allogeneic HCT

Since patients in MRD-positive remission have worse outcomes even if they convert to MRD-negativity after allografting [63,64,80], it has been hypothesized that MRD eradication before allogeneic HCT may be of benefit. One proposed approach entails additional or intensified chemotherapy [81]. A recent randomized trial showed improved outcomes with treatment intensification in older adults who achieved an MRD-positive remission after the first cycle of standard induction chemotherapy [82], but the impact in the subset of patients subsequently allografted was not reported. Notably, many of the studies on pre-emptive “MRD eraser” therapies completed to date have focused on *NPM1*-mutated AML [44,83,84,85,86]. While these non-randomized studies established the feasibility of this therapeutic strategy, firm data regarding longer-term benefits are lacking. Likewise, the roles that molecularly targeted drugs (e.g., *FLT3*-, *IDH*-, or menin inhibitors) play when given to patients before HCT to eradicate MRD are largely unknown.

Administering “MRD eraser” therapy before allogeneic HCT requires careful weighing of potential benefits vs. toxicities, especially because such treatments may not only delay time to allografting but could also introduce significant immediate complications that increase NRM. Moreover, additional courses of treatment may promote clonal evolution and the emergence of mutations that increase the risk of AML relapse post-HCT [87].

### 5.3. Selection of Conditioning Intensity

Several studies, including randomized trials, suggested lower relapse rates and, in some cases, improved outcomes with MAC compared to reduced-intensity (RIC) or non-myeloablative (NMA) conditioning [88,89,90,91,92,93,94,95]. Thus, the relationship between conditioning intensity and post-HCT outcomes of patients with or without MRD has garnered significant attention. In a post-hoc analysis of a subset of the patients in the large prospective randomized BMT CTN 0901 trial, MAC did mitigate the poor prognosis associated with pre-HCT NGS MRD-positivity but did not significantly improve outcomes relative to RIC in patients without MRD [96]. Partially consistent with these findings, a retrospective analysis by the European Society of Blood and Marrow Transplantation (EBMT) showed a reduced incidence of relapse and improved relapse-free survival with MAC relative to RIC in AML patients with pre-HCT MRD younger than age 50, but no benefit above age 50 [97]. In contrast, in other retrospective and prospective studies, including the randomized NCRI FIGARO trial, no relative benefit of intensified conditioning was seen for patients with pre-HCT MRD [63,98,99,100]. Altogether, these studies support the use of MAC in suitable patients with pre-HCT MRD, with the caveat that there remains uncertainty regarding the magnitude of the benefit of MAC relative to RIC or NMA conditioning.

### 5.4. Choice of Stem Cell Source

While HLA-matched related or unrelated donors are usually considered the first choice as donors for allogeneic HCT in AML, other donor types including HLA-mismatched/haploidentical donors or umbilical cord blood (UCB) are established and effective alternatives, each with their own advantages and drawbacks [101,102,103]. Limited data from retrospective studies indicate relapse rates for AML patients with pre-HCT MRD may be lower, and survival possibly better, following HLA-haploidentical or UCB HCT compared to HLA-matched donor HCT [104,105,106,107,108,109]. These findings raise the possibility of stronger GVL effects with HLA-haploidentical or UCB allografts, which may be particularly beneficial in patients with pre-HCT MRD, but further prospective, well-controlled studies are necessary before drawing definitive conclusions. Conversely, while autologous HCT for AML has been used less frequently in recent years [110,111], several recent studies suggested potential benefits in patients with favorable- or intermediate-risk AML in MRD-negative remission relative to post-remission chemotherapy [112,113].

### 5.5. Modulation of Immunosuppressive Therapy

As the curative potential of allogeneic HCT largely depends on immune-mediated GVL effects from donor T cells, manipulation of immunosuppressive therapy has long been utilized to treat relapsed/refractory AML after HCT [114,115]. In the setting of morphologic relapse with rapid disease progression, however, the likelihood of response with withdrawal of immunosuppression alone is generally very low, and additional therapies are typically needed [115]. This may be different for patients with MRD-level disease. For instance, for patients with MRD prior to allografting, the earlier taper of immunosuppressive drugs to increase GVL effects is considered an intervention [116]; still, firm data on the efficacy of this approach are lacking, and there is no consensus on the best approach or timing to post-HCT immunosuppression therapy taper in these patients. In patients with post-HCT MRD, early withdrawal of immunosuppressive therapy may lead to durable remission [117,118].

### 5.6. Preemptive Use of DLI

Currently, predictors of response to DLI, another common immunotherapy intervention to manage AML relapse after allogeneic HCT, are poorly established, however, increasing evidence suggests that MRD status may predict the benefit of DLI [67,119]. Spurred by the idea that DLI would have a greater effect with lower leukemia burden, early data showed comparable survival between patients with post-HCT MRD who were treated with DLI and MRD-negative patients, while outcomes were significantly worse in patients with MRD who did not receive DLI [120]. In more recent retrospective analyses, DLI given at the time of detection of MRD was shown to convert patients to MRD-negativity and to improve long-term survival [67,119]. For instance, one study observed no apparent improvement in outcome among 23 patients who received DLI while in MRD-negative remission whereas 73% of the 15 patients in MRD-positive remission and 32% of the 38 patients with overt leukemia at the time of DLI converted to MRD-negative or achieved remission within 90 days of DLI, respectively [67]. As MRD status at the time of DLI was prognostic for relapse and relapse-free survival, available data suggests benefit of DLI in these patients [67]. A prospective non-randomized study evaluating risk-stratified use of DLI with or without IL-2 in patients with standard-risk leukemia who developed MRD-positivity after allografting found that these patients had similar relapse rates to those who tested MRD-negative at all time points after HCT, further supporting the benefit of DLI in patients with MRD [121]. With DLI, the risk of graft-versus-host disease (GVHD) remains a significant concern and may limit any benefit, especially when used preemptively. Whether the combination of DLI with agents like azacitidine could further augment the GVL response against AML while mitigating GVHD is currently unknown [122].

### 5.7. Post-HCT Maintenance Therapy

There has been a long-standing interest in post-HCT maintenance therapy to reduce the risk of relapse–a concept of particular importance for patients with pre-HCT MRD [123]. Initial efforts focused on hypomethylating agents. To date, the effectiveness of these agents in enhancing GVL effects remains unclear, with conflicting results from several non-randomized prospective studies [123,124,125,126]. More recently, attention has shifted toward molecularly targeted therapies, including tyrosine kinase inhibitors (TKIs). The demonstrated benefits of TKIs as part of non-transplant therapy for *FLT3*-mutated AML [127,128,129] generated interest in their use as pre-emptive maintenance therapies after allogeneic HCT. Data derived not only from uncontrolled studies but also from several randomized trials showed reduced relapse rates and longer survival with TKIs [100,130,131,132]. Further prospective studies are essential to clarify whether/which distinct patient subsets derive consistent benefits from such therapies, with the goal of limiting the use of TKIs to individuals most likely to benefit while avoiding unnecessary toxicities in others. In the MORPHO trial, for example, improved relapse-free survival with gilteritinib was restricted to patients with pre- or post-HCT *FLT3*-ITD MRD [100]. Other targeted therapeutics of interest include inhibitors of mutant *IDH* (*mIDH*) and *menin*, which have anti-AML efficacy as monotherapy or in combination with lower-intensity therapy [133,134,135,136]. Early phase trials with *mIDH1* and *mIDH2* inhibitors demonstrate these drugs are safe, well tolerated, and potentially effective as maintenance therapy post-HCT [137,138,139], but it remains to be determined whether there is unique value as MRD-directed therapeutic intervention.

### 5.8. Treatment of MRD Relapse After Allogeneic HCT

The strong link between the re-emergence or rising levels of MRD over time with overt disease recurrence [73,140,141] provides a compelling rationale to initiate salvage therapy early when the patient has evidence of molecular relapse but is still in morphologic remission. Several primarily retrospective non-transplant studies suggest that AML therapies (e.g., arsenic trioxide [for APL] and azacitidine) can be highly effective when given before morphologic relapse and may lead to improved outcomes [85,86,141,142,143,144]. Data supporting the use of salvage therapies in the post-HCT setting remain sparse; in the non-randomized phase 2 RELAZA2 trial, initiation of azacitidine for post-HCT MRD was safe and increased the interval from MRD detection to morphologic relapse compared to historical controls [145]. However, such data must be interpreted with caution considering multiple confounding factors including patient selection and lead-time bias. Prospective, controlled studies will be necessary to assess whether treatment before morphologic relapse indeed improves outcomes, and what toxicities might result from early interventions. As some patients with MRD-re-emergence or increasing levels of MRD may not develop overt disease, the potential for early interventions to expose some patients to unnecessary and potentially toxic AML therapy is a forefront concern. Moreover, given the wide variation in relapse dynamics even among genetically similar leukemias, the ideal approach for the use and timing of serial MRD testing to detect early relapse remains unknown. Likewise, it remains unclear whether serially negative MRD tests could inform decisions to withhold further treatment or alter therapeutic strategies.

### 5.9. Conclusions on MRD to Guide Therapy Related to Allogeneic HCT

The concept of risk-stratified therapy based on MRD status is simple, attractive, and intuitive. This approach might optimize the balance between treatment efficacy and toxicity to improve patient outcomes. However, it is not without caveats. Given the limitations of MRD testing and interpretation, MRD-directed therapy requires careful evaluation: intensified treatments may result in increased toxicities and subsequent morbidity/mortality, potentially preventing further (potentially curative) therapies down the line. On the other hand, therapy de-intensification could lead to undertreatment and AML relapse. Current evidence from large randomized or non-randomized prospective studies on MRD-directed therapy remains scarce and conflicting [82,112,146,147]. Further research is required to understand whether MRD indeed represents a meaningful prognostic target or whether it merely is an indicator of inherently adverse disease biology and poor outcomes regardless of MRD-directed approaches.

## 6. MRD for Monitoring After Allogeneic HCT

Although there is no established standard for MRD monitoring after allo-HCT, expert panels have provided guidance on the frequency, sources, and methodology of MRD monitoring after HCT [3,8]. The ELN MRD Working Party recommends that MRD assessments be conducted at critical junctures before HCT (i.e., at the completion of consolidation therapy and before transplant) and at regular intervals post-HCT (i.e., 3 to 6-month intervals for up to 2 years), with continued MRD monitoring beyond 2 years based on individual clinical features and treatment goals. The choice of tissue source and methodology for MRD testing is influenced by the genetic profile of the patient’s AML. In general, MFC on bone marrow specimens is recommended as bone marrow provides greater sensitivity than blood and is better validated when MRD testing is done for prognostic purposes. On the other hand, peripheral blood monitoring has proven useful for molecular MRD testing (e.g., *NPM1*, *FLT3*-ITD, *CBF* translocations, *PML-RARA*, among others). Regardless of the presence of specific molecular markers, concurrent assessment with MFC and molecular tests may be useful in tracking clonal evolution/diversity. Further studies to refine monitoring schemes, possibly tailored to the biology of individual AMLs, are needed. For example, in a recent retrospective study, the majority of patients had evidence of MRD by molecular testing on peripheral blood samples around 1–2 months prior to overt relapse, with a minority with peripheral blood MRD-positivity at 3 months, suggesting that a much shorter monitoring interval may be required [66]. This study additionally highlighted the need to consider that many mutations demonstrate clonal instability from diagnosis to relapse, with specific classes of leukemia genes more suitable as stable longitudinal disease markers [66].

## 7. MRD as Efficacy/Response Biomarker in Patients Considered for Allogeneic HCT

As standard endpoints in AML trials may take years and require a large “number needed to treat” for statistical analyses, there is rising interest in using MRD as an efficacy-response surrogate biomarker to accelerate drug development and/or approval for new AML therapies. While MRD assessments have been accepted by regulatory authorities as a surrogate endpoint in other hematologic malignancies, use in AML remains challenging. Guidance documents from the U.S. Food and Drug Administration (FDA) outline the regulatory considerations for MRD as a surrogate efficacy-response biomarker, including biological plausibility, demonstration of its prognostic value in epidemiological studies, and evidence of its predictive value in clinical trials [148,149]. While many studies, including findings from a large meta-analysis [52,53], address the first two requirements, evidence from randomized clinical trials to evaluate its possible predictive value remains limited. Data from a randomized phase 3 trial testing oral azacitidine vs. placebo as maintenance therapy showing that a significant number of patients with MRD at baseline converted to MRD-negativity in the placebo arm highlights the challenges the field faces in using MRD as an efficacy-response biomarker for AML [150]. Results from several ongoing prospective trials evaluating treatment effects on both MRD and survival are eagerly awaited to address this unmet need.

## 8. Conclusions and Future Perspective

MRD testing has become routine for patients with AML considered for allogeneic HCT. Robust data demonstrating MRD before or after allografting identifies a subset of patients at particularly high risk of relapse, and poor survival validates its use as a prognostic biomarker to estimate the likelihood (and timing) of leukemia relapse. Collaborative efforts to standardize and optimize MRD methodologies are ongoing. However, while serial MRD monitoring may allow early relapse detection and timely intervention, to what degree (if any) MRD monitoring improves outcomes requires further evaluation. Likewise, although emerging data suggest benefits, further well-controlled studies will need to clarify whether MRD-directed treatment changes (intensification, deintensification, or cessation) before, during, or after allografting improve outcomes and if it will find a firm place in AML treatment guidelines. Fortunately, many larger efforts are underway that test the value of MRD not only as a prognostic but also as a predictive biomarker for treatment decision-making. The importance of including contemporary MRD assays in current and future trials to expand our existing knowledge to refine expert consensus and recommendations cannot be overstated.

## Figures and Tables

**Table 1:** Methodologies to assess AML cell burden.

Test	Method	Sensitivity	Advantages	Disadvantages
Morphology	Microscopic examination of leukemic cells in bone marrow	~10^−2^	Availability Applicable to all AML cases	Low sensitivity Unable to reliably distinguish normal from abnormal myeloblast by light microscopy
Multiparameter Flow Cytometry (MFC)	Detection of immunophenotypically abnormal cell populations based on surface/intracellular markers using fluorescent antibodies	10^−3^–10^−5^	Wide applicability (>90% of AML cases) Relatively high sensitivity Rapid turn-around time Assessment of hemodilution Distinguishes between live and dead cells Identification of immunotherapy targets	Expertise and experience required Fresh sample preferred Variability in methodology and performance characteristics Not all AMLs have an abnormal immune phenotype
Quantitative PCR (qPCR)	Quantification of single molecular fusion transcripts and mutations	10^−5^–10^−6^	High sensitivity Assays for some mutations well validated (e.g., *NPM1*, *RUNX1*-*RUNX1T1*, and *CBFB*-*MYH11*) Well standardized	More limited applicability to subsets of patients Requires well-delineated mutation targets Some abnormalities detected may not be prognostic
Next-Generation Sequencing (NGS)	Quantification of multiple molecular abnormalities	10^−5^–10^−6^	Wide applicability (>90% of AML cases) Identification of immunotherapy targets High sensitivity achievable	Sequencing errors can cause both false negatives and positives unless the error correction method included Not standardized Some mutations are not prognostic (e.g., *DNMT3A*, *ASXL1*, and *TET2*)

## Data Availability

Not applicable.
